# Supplementary material for: Identification of Transferrin Receptor 1 (TfR1) Overexpressed in Lung Cancer Cells, and Internalization of Magnetic Au-CoFe2O4 Core-Shell Nanoparticles Functionalized with Its Ligand in a Cellular Model of Small Cell Lung Cancer (SCLC)
Source: Pharmaceutics. 2022 Aug 17;14(8):1715. doi: 10.3390/pharmaceutics14081715 (PMC9413248; doi:10.3390/pharmaceutics14081715)
Supplement: Supplementary file 1 [file pharmaceutics-14-01715-s001.zip › SuppMat.pdf]

Supplemental Information

Label-Free Proteomic Analysis of Enriched Membranes Revealed Transferrin-1 Receptor as a Potential Target for Lung Cancer using Functionalized Core–Shell Nanoparticles

Rocío Villalobos-Manzo[a], Emmanuel Ríos-Castro[b], José Manuel Hernández-Hernández[c] and José Tapia-Ramírez[a*]

[a]Departamento de Genética y Biología Molecular, Cinvestav-IPN, Ciudad de México, México, C.P. 07360.

[b]Unidad de Genómica, Proteómica y Metabolómica (UGPM), LaNSE, Cinvestav-IPN, Ciudad de México, México, C.P. 07360.

[c]Departamento de Biología Celular, Cinvestav-IPN, Ciudad de México, México, C.P. 07360.

*Corresponding Author: Dr. José Tapia-Ramírez; E-mail: jtapia@cinvestav.mx

Keywords: Label-free, Mass Spectrometry, Nanoparticles, Small Cell Lung Cancer (SCLC), Transferrin Receptor (CD71 receptor).

Figure S1. Peptide reliability and confidence. **A)** Histogram representing a total of 123,724 detected peptides; 86.92% of them fall into an error maximum of ± 10 ppm, indicating that the calibration of the mass spectrometer was adequate. **B)** Pie chart representing the types of peptides identified, most of them, 79.1%, are peptides of high quality denominated PepFrag1, these peptides were the most reliable in the study since were identified during database search pass-1 by the algorithm of Progenesis software , 6% represent missed cleavage peptides, which indicate that the enzymatic activity of trypsin was efficient, 10 % were peptides with variable modifications including PTMs (VarMod), 3.8% are considered PepFrag2 which represent peptides with less reliability because they were identified with less restrictions during database search, and finally, neutral loss of H_2O and NH_3 as well as fragmented peptides at ion source (InSource ions) represent <1% of total peptides. **C)** Most of PepFrag1 peptides are concentrated at a maximum of ± 10 ppm throughout the analyzed *m / z* range (black dots), besides these peptides are condensed mostly in *m/z* 500-900, which is expected in proteomic experiments. **D)** Ion mobility vs m/z plots. Plot which represents the way in which ions (only peptides) are moving inside of the mobility cell of Synapt G2-S*i* after removing ions z=1^+ based on their drift times; green dots, ions z=2^+, red dots, ions z=3^+, other colors, ions with superior charge states. These ions (123,724 peptides) were used to identification and quantitation in $UDMS^E$ mode.

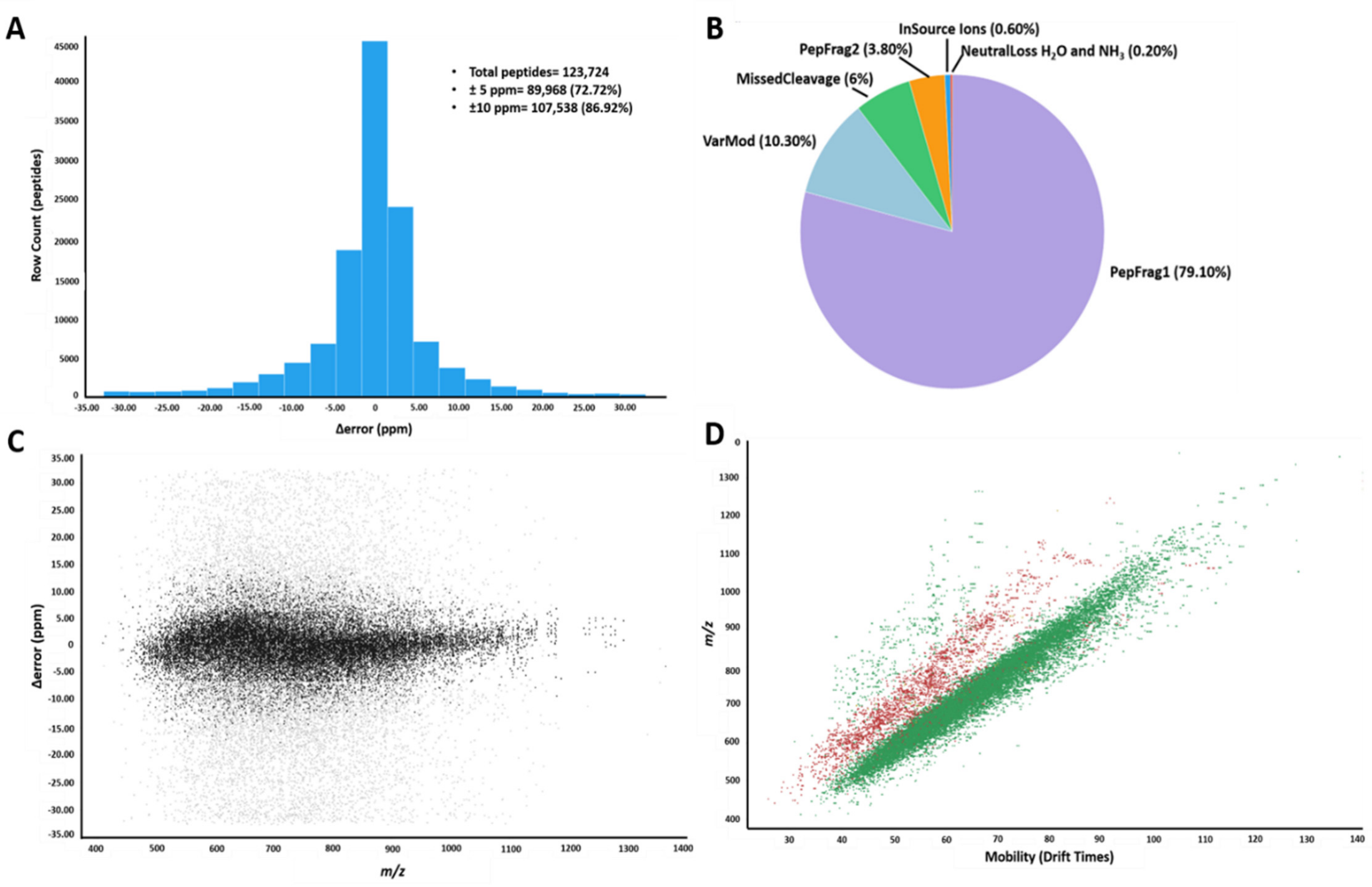

Figure S2. Protein reliability and confidence. **A)** Dynamic range of quantified membrane proteins (1,298). In magenta, proteins of MRC5 cell line and in green, proteins of H69AR cell lines. Abscissa axis corresponds to the number quantified proteins (ID´s); ordinate axis corresponds to the average of Hi3 intensities for each detected protein (values are represented as base 10 logarithm). **B)** Volcano plot representing all filtered proteins; gray circles unchanged proteins (186), red circles upregulated proteins (188), green circles downregulated proteins (174). Abscissa axis corresponds to the p-value of each detected protein in the technical triplicate (values represented as -Log10) and ordinate axis corresponds to the ratio of the average of Hi3 intensities (H69AR/MRC5) for each detected protein (values are represented as base 2 logarithm).

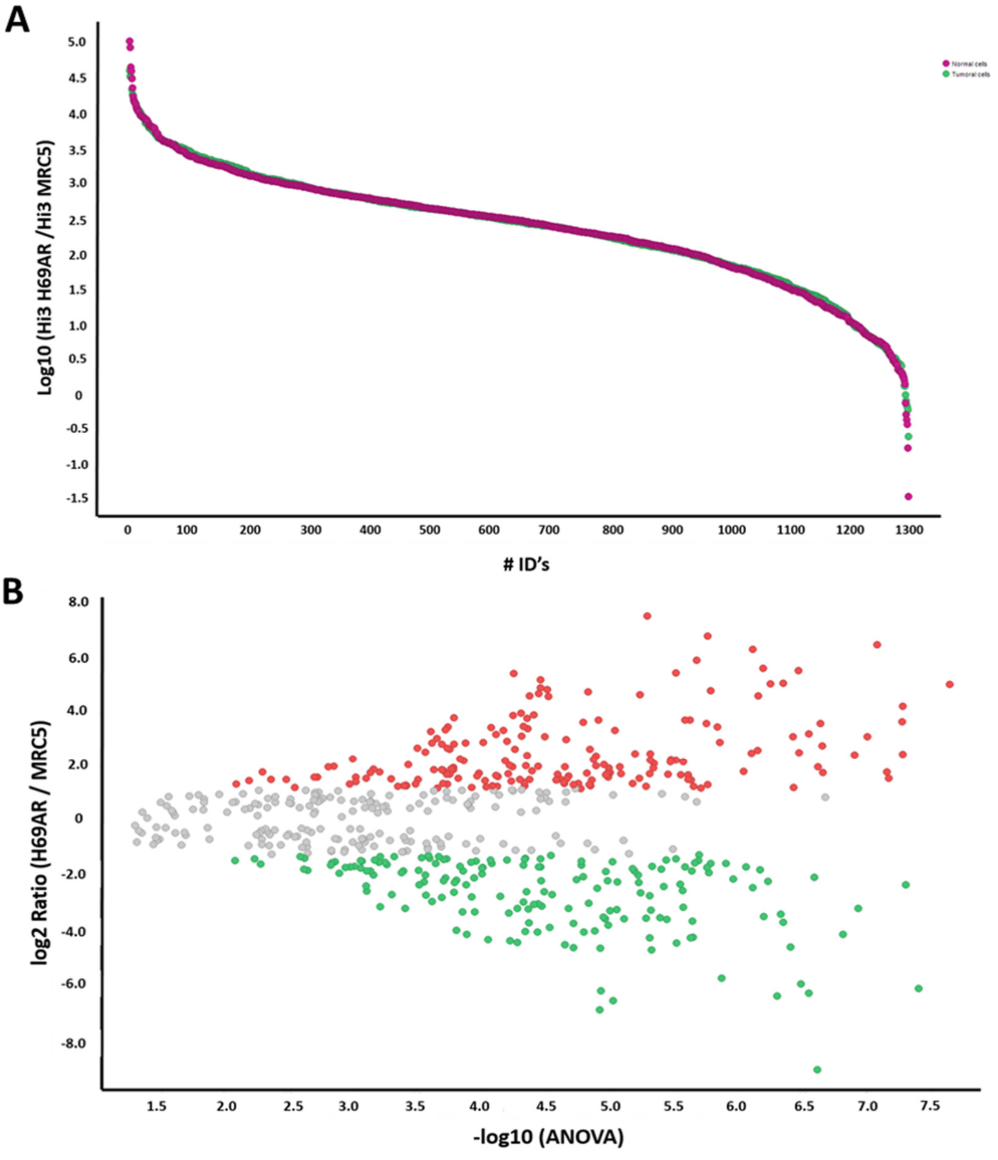

Figure S3. Heatmap representation of relative abundance of 548 filtered membrane proteins for both cell lines. Triplicates for both conditions were used to generate the plot. Relative abundance is represented using “Z-Score”. Marked differences in expression were revealed between both cell lines.

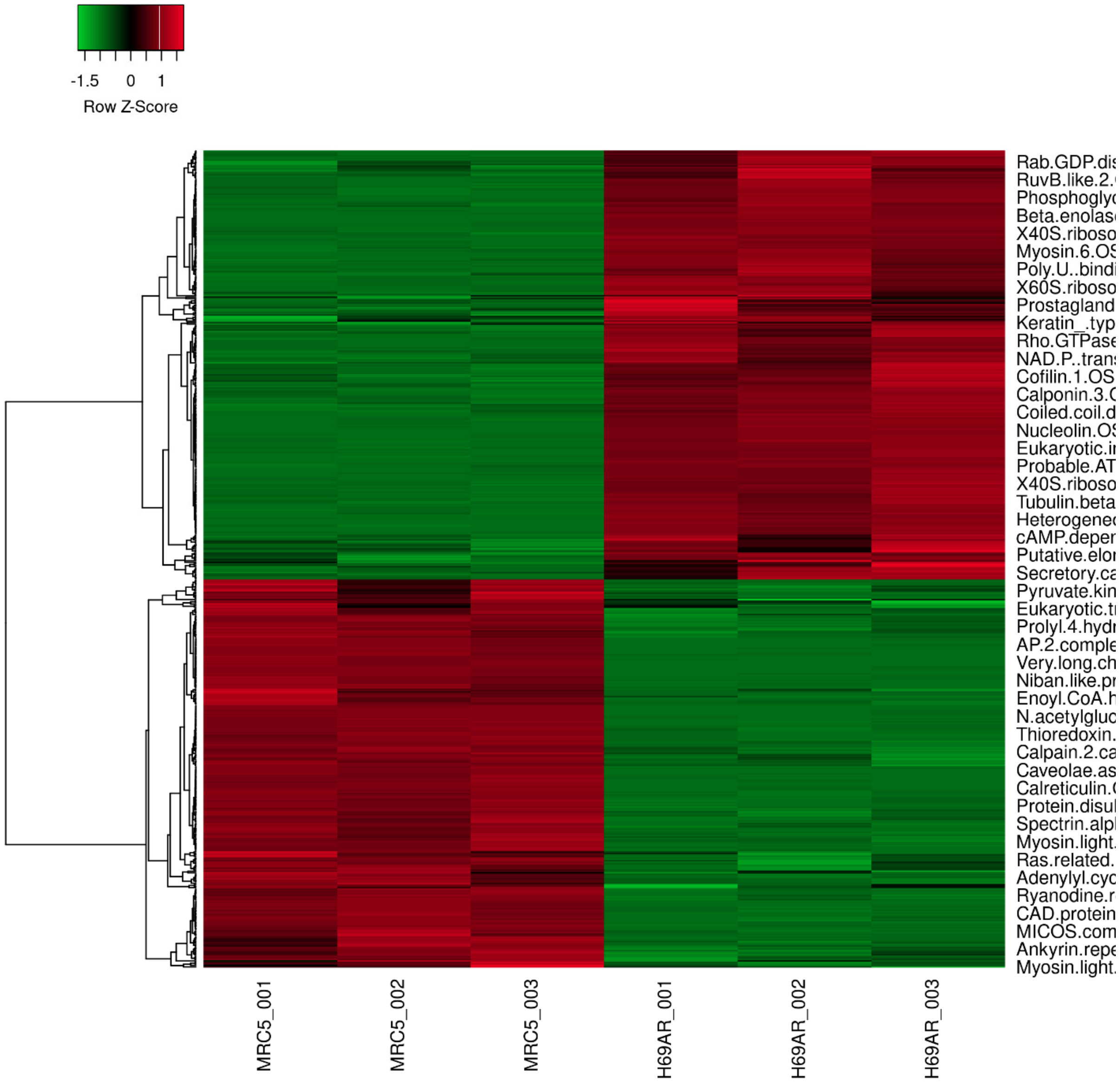

Figure S4. Differentially expressed proteins interactome. A highly interconnected network has been generated in STRING using 427 differentially expressed proteins; the effect that they exert at molecular level is differentiated in both cell lines since the abundance of each protein in a cell line is contrary in the other. The interactome reveals the importance of these proteins in the metabolism of both cell lines with the main emphasis on cell proliferation since many of them participate in specific processes such as cell cycle (blue spheres), protein synthesis (red spheres), immune response (yellow spheres) and vesicular trafficking (green spheres). Among them we highlight TFRC (black circle), which has a high degree of interconnection that impact in downstream actions.

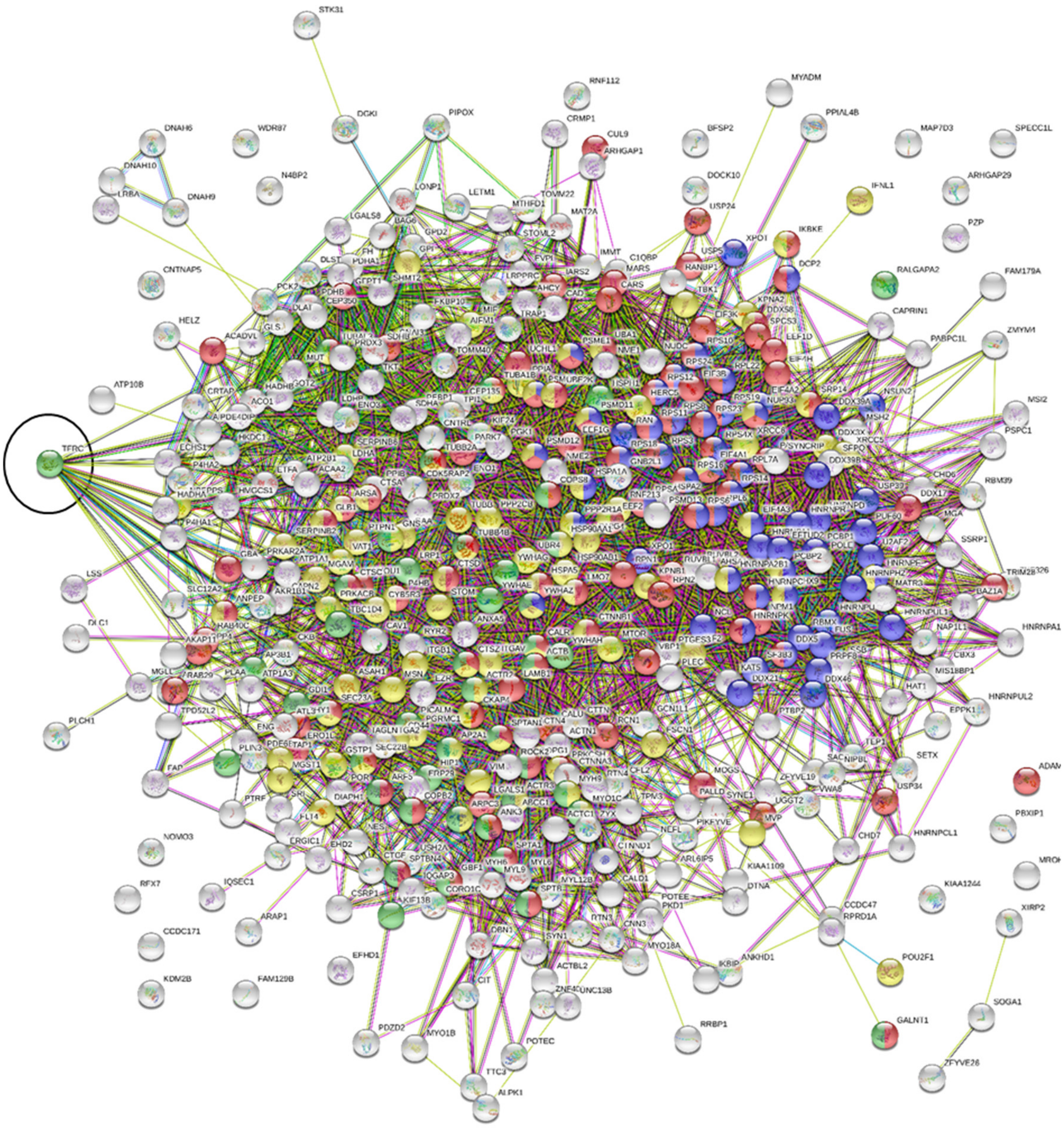

Table S1. Differentially expressed proteins. Through the label-free analysis we were able to report 188 proteins up-regulated in membranes of H69AR cells and 174 in membranes of MRC5 cells. Also, 32 and 33 proteins were reported as exclusive in membranes of H69AR and MRC5 cells respectively.

Table S2. Identified proteins by mass spectrometry. On the first sheet "Total Proteins" are all proteins detected in the experiment, the raw intensities measured were provided. On the second sheet "Only ID proteins" are proteins which were not quantified, because they have not unique peptides. On the third sheet, "Exclusive Proteins" are proteins which are unique either on MRC5 or H69AR. On the fourth sheet "Quantified Proteins" are all shared proteins in both conditions (1,298), which could be quantified by the Hi3 method. On the fifth sheet "Unchanged", we reported 186 proteins which did not change the abundance between MRC5 and H69AR cell lines. On the sixth sheet "Up", we reported 188 proteins which are up-regulated in H69AR cell line; and finally, on the seventh sheet "Down", we reported 174 proteins which are down-regulated in H69AR cell line. “Unchanged”, “Up”, “Down” proteins were selected through a restrictive filter: a coefficient of variation (CV) ≤ 0.30, at least 2 peptides per protein, considering in those at least 1 unique peptide and ANOVA ≤ 0.05 and that they have been detected in all triplicates.

Table S3. Differentially expressed proteins participate in 212 biological pathways according to the analysis carried out in Reactome. All reported biological pathways have a False Discovery Rate (FDR) ≤0.05.
