# Supplementary material for: Identification of Transferrin Receptor 1 (TfR1) Overexpressed in Lung Cancer Cells, and Internalization of Magnetic Au-CoFe2O4 Core-Shell Nanoparticles Functionalized with Its Ligand in a Cellular Model of Small Cell Lung Cancer (SCLC)
Source: Pharmaceutics. 2022 Aug 17;14(8):1715. doi: 10.3390/pharmaceutics14081715 (PMC9413248; doi:10.3390/pharmaceutics14081715)
Supplement: Supplementary file 1 [file pharmaceutics-14-01715-s001.zip › Sup_Table S1.pdf]

Proteins up-regulated in H69AR cell line					
**Accession**	**Description**	**Peptide Count**	**Unique peptides**	**Confidence score**	**Max Fold change**
Q9C0G6	Dynein heavy chain 6_ axonemal	29	1	108.37	Exclusive in H69AR
P58107	Epiplakin	23	1	73.87	Exclusive in H69AR
Q7Z333	Probable helicase senataxin	17	1	63.52	Exclusive in H69AR
P07196	Neurofilament light polypeptide	6	4	45.06	Exclusive in H69AR
Q6ZQQ6	WD repeat-containing protein 87	12	1	36.39	Exclusive in H69AR
P98161	Polycystin-1 OS=Homo sapiens	7	1	32.84	Exclusive in H69AR
Q6TFL3	Coiled-coil domain-containing protein 171	8	1	30.99	Exclusive in H69AR
E9PD68	Dihydropyrimidinase-related protein 1	5	1	30.50	Exclusive in H69AR
Q8IZP2	Putative protein FAM10A4	5	1	28.75	Exclusive in H69AR
Q8IWI9	MAX gene-associated protein	8	2	26.96	Exclusive in H69AR
A0A0D9SFK2	Unconventional myosin-XVIIIa	7	1	25.05	Exclusive in H69AR
Q14498	RNA-binding protein 39	4	2	19.58	Exclusive in H69AR
Q66GS9	Centrosomal protein of 135 kDa	4	1	17.41	Exclusive in H69AR
A0A087X1B2	U4/U6.U5 tri-snRNP-associated protein 2	3	1	16.39	Exclusive in H69AR
Q9P0Z9	Peroxisomal sarcosine oxidase	3	1	14.86	Exclusive in H69AR
Q9NR30	Nucleolar RNA helicase 2 OS=Homo sapiens	3	1	14.73	Exclusive in H69AR
O94823	Probable phospholipid-transporting ATPase VB	4	1	14.59	Exclusive in H69AR
Q13515	Phakinin	4	1	13.60	Exclusive in H69AR
Q01581	Hydroxymethylglutaryl-CoA synthase_ cytoplasmic	3	1	12.72	Exclusive in H69AR
Q96S21	Ras-related protein Rab-40C	3	1	12.55	Exclusive in H69AR
A0A0C4DGQ6	Regulation of nuclear pre-mRNA domain-containing protein 1A	2	1	11.97	Exclusive in H69AR
Q69YQ0	Cytospin-A	4	1	11.83	Exclusive in H69AR

G3V438	Activator of 90 kDa heat shock protein ATPase homolog 1 (Fragment)	2	1	11.02	Exclusive in H69AR
Q5TH69	Brefeldin A-inhibited guanine nucleotide-exchange protein 3	3	1	9.91	Exclusive in H69AR
B4DWR3	Prefoldin subunit 3	2	1	9.70	Exclusive in H69AR
G3V2D8	Zinc finger FYVE domain-containing protein 26	3	1	8.34	Exclusive in H69AR
Q5BKZ1	DBIRD complex subunit ZNF326	2	1	7.83	Exclusive in H69AR
P62266	40S ribosomal protein S23	2	1	7.71	Exclusive in H69AR
Q9ULX5	RING finger protein 112	2	1	7.38	Exclusive in H69AR
B1ANM0	Galectin (Fragment)	2	1	6.57	Exclusive in H69AR
K7EJ84	Dystrobrevin alpha (Fragment)	1	1	6.08	Exclusive in H69AR
P58397	A disintegrin and metalloproteinase with thrombospondin motifs 12	2	1	6.03	Exclusive in H69AR
P07910-2	Isoform C1 of Heterogeneous nuclear ribonucleoproteins C1/C2	12	1	102.95	187.47
P07910	Heterogeneous nuclear ribonucleoproteins C1/C2	16	3	95.29	113.10
P52292	Importin subunit alpha-1	7	3	43.23	89.93
O14929	Histone acetyltransferase type B catalytic subunit	3	2	15.85	80.34
F8W6I7	Heterogeneous nuclear ribonucleoprotein A1	9	2	78.38	61.31
K7ENG2	U2 snRNP auxiliary factor large subunit	3	2	19.67	49.85
P43243	Matrin-3	11	7	69.95	46.94
O43390	Heterogeneous nuclear ribonucleoprotein R	15	9	112.75	44.40
E9PGT6	COP9 signalosome complex subunit 8	2	1	12.37	43.70
Q9NS69	Mitochondrial import receptor subunit TOM22 homolog	2	2	12.48	36.95
Q15393	Splicing factor 3B subunit 3	11	6	44.37	34.17
P33527	Multidrug resistance-associated protein 1	63	50	381.14	33.49
P02786	Transferrin receptor protein 1	28	18	173.00	32.86
P23246	Splicing factor_ proline- and glutamine-rich	7	6	45.73	30.12
P30626	Sorcin	6	5	29.52	29.06
P22626	Heterogeneous nuclear ribonucleoproteins A2/B1	26	14	141.98	28.05

Q08211	ATP-dependent RNA helicase A	18	9	107.40	27.24
P20020	Plasma membrane calcium-transporting ATPase 1	12	3	59.28	26.25
O14980	Exportin-1	10	7	61.64	25.29
O43592	Exportin-T	6	2	32.56	24.58
O60812	Heterogeneous nuclear ribonucleoprotein C-like 1	8	1	50.00	24.50
P12277	Creatine kinase B-type	19	14	100.42	24.28
A0A1W2PQ51	Probable ATP-dependent RNA helicase DDX17	14	3	92.13	18.95
Q9Y230	RuvB-like 2	7	5	42.68	15.77
A0A0J9YVP6	Poly(U)-binding-splicing factor PUF60 (Fragment)	3	2	17.61	15.19
Q96QB1	Rho GTPase-activating protein 7	13	2	51.83	15.08
P16435	NADPH--cytochrome P450 reductase	7	2	44.57	14.16
Q1KMD3	Heterogeneous nuclear ribonucleoprotein U-like protein 2	7	2	28.76	13.85
Q32P51	Heterogeneous nuclear ribonucleoprotein A1-like 2	17	5	63.56	13.38
P38159	RNA-binding motif protein_ X chromosome	5	2	33.69	13.29
P35637	RNA-binding protein FUS	6	4	30.62	13.25
P13929	Beta-enolase	8	1	47.83	12.77
C9JDM3	Ran-specific GTPase-activating protein (Fragment)	2	2	15.43	12.56
P55011	Solute carrier family 12 member 2	9	3	40.26	12.16
Q13838	Spliceosome RNA helicase DDX39B	9	3	65.69	12.12
P61086	Ubiquitin-conjugating enzyme E2 K	6	1	40.68	11.28
O00148	ATP-dependent RNA helicase DDX39A	17	6	73.50	11.22
Q9Y266	Nuclear migration protein nudC	6	3	35.00	11.19
Q9UKA9	Polypyrimidine tract-binding protein 2	3	2	19.89	11.03
Q92616	eIF-2-alpha kinase activator GCN1	21	6	80.91	10.69
P17844	Probable ATP-dependent RNA helicase DDX5	20	9	90.79	10.37
Q9Y265	RuvB-like 1	8	6	40.61	10.30
P52597	Heterogeneous nuclear ribonucleoprotein F	10	5	56.73	10.23
O15018	PDZ domain-containing protein 2	9	3	27.87	9.97
O00264	Membrane-associated progesterone receptor component 1	3	2	19.95	9.33

P38919	Eukaryotic initiation factor 4A-III	8	3	50.04	9.28
P23526	Adenosylhomocysteinase	6	2	28.93	8.85
P19338	Nucleolin	33	23	168.76	8.73
Q00839	Heterogeneous nuclear ribonucleoprotein U	13	9	86.47	8.69
Q8WXF1	Paraspeckle component 1	3	3	19.05	8.69
Q9UNM6	26S proteasome non-ATPase regulatory subunit 13	5	3	28.53	8.32
A0A087WVC4	cAMP-dependent protein kinase catalytic subunit beta	4	1	20.52	8.16
Q63HN8	E3 ubiquitin-protein ligase RNF213	28	3	106.67	7.73
A0A024RCR6	HLA-B associated transcript 3_ isoform CRA_a	3	2	15.52	7.58
B4DHE8	cDNA FLJ56904_ highly similar to RNA-binding protein Musashi homolog 2	5	3	32.26	7.56
A0A087WXQ7	POTE ankyrin domain family member C	2	1	10.94	7.48
Q92538	Golgi-specific brefeldin A-resistance guanine nucleotide exchange factor 1	2	1	10.40	7.45
Q07021	Complement component 1 Q subcomponent-binding protein_ mitochondrial	4	1	24.42	7.19
P43246	DNA mismatch repair protein Msh2	7	2	35.87	7.18
P06748	Nucleophosmin	10	8	78.67	6.94
P13533	Myosin-6	10	1	37.30	6.56
Q10472	Polypeptide N-acetylgalactosaminyltransferase 1	6	3	33.57	6.50
A0A087WYT3	Prostaglandin E synthase 3	2	1	9.18	6.32
P15531	Nucleoside diphosphate kinase A	15	4	72.22	6.29
P62826	GTP-binding nuclear protein Ran	12	9	56.82	6.28
P30153	Serine/threonine-protein phosphatase 2A 65 kDa regulatory subunit A alpha isoform	7	5	44.64	6.15
Q9NQT8	Kinesin-like protein KIF13B	9	2	42.97	5.88
P61978	Heterogeneous nuclear ribonucleoprotein K	19	15	96.51	5.77
P62258	14-3-3 protein epsilon	15	9	121.45	5.67
P05455	Lupus La protein	5	2	30.27	5.61
Q14974	Importin subunit beta-1	18	16	106.06	5.60
P08238	Heat shock protein HSP 90-beta	68	25	399.63	5.52

P13010	X-ray repair cross-complementing protein 5	8	4	43.33	5.47
Q96BY6	Dedicator of cytokinesis protein 10	9	3	30.38	5.40
O00571	ATP-dependent RNA helicase DDX3X	14	6	91.36	5.34
P37108	Signal recognition particle 14 kDa protein	2	2	13.95	4.95
Q02878	60S ribosomal protein L6	3	3	18.77	4.94
P84085	ADP-ribosylation factor 5	6	3	30.83	4.86
P06733	Alpha-enolase	33	20	180.33	4.83
Q15366	Poly(rC)-binding protein 2	8	4	33.44	4.79
P31153	S-adenosylmethionine synthase isoform type-2	3	1	15.31	4.78
Q15029	116 kDa U5 small nuclear ribonucleoprotein component	9	6	50.35	4.72
A0A087X054	Hypoxia up-regulated protein 1	13	6	71.24	4.72
Q9UQ80	Proliferation-associated protein 2G4	8	3	43.33	4.62
P69849	Nodal modulator 3	13	9	81.60	4.56
Q12931	Heat shock protein 75 kDa_ mitochondrial	13	8	91.17	4.39
Q9UI47	Catenin alpha-3	5	1	23.70	4.28
Q9P2D1	Chromodomain-helicase-DNA-binding protein 7	19	5	66.39	4.28
O00231	26S proteasome non-ATPase regulatory subunit 11	9	6	54.48	4.23
O95197	Reticulon-3	11	4	47.85	4.17
P12956	X-ray repair cross-complementing protein 6	19	9	92.60	4.14
P11586	C-1-tetrahydrofolate synthase_ cytoplasmic	12	3	59.77	4.08
P56192	Methionine--tRNA ligase_ cytoplasmic	9	3	55.65	4.08
A0A087WTT1	Polyadenylate-binding protein	10	3	72.79	4.05
D6RAF8	Heterogeneous nuclear ribonucleoprotein D0 (Fragment)	7	3	47.86	4.04
P62701	40S ribosomal protein S4_ X isoform	6	6	42.46	4.03
P25398	40S ribosomal protein S12	2	2	6.11	4.01
Q7L014	Probable ATP-dependent RNA helicase DDX46	5	2	19.12	3.99
Q9UJZ1	Stomatin-like protein 2_ mitochondrial	5	3	32.02	3.96
P0DMV8	Heat shock 70 kDa protein 1A	19	4	101.31	3.90
P14174	Macrophage migration inhibitory factor	2	1	13.52	3.90
Q9Y2I7	1-phosphatidylinositol 3-phosphate 5-kinase	15	3	52.59	3.80

E7EP17	Dynein heavy chain 9_ axonemal	7	1	25.66	3.78
K7ERF1	Eukaryotic translation initiation factor 3 subunit K	2	1	12.19	3.76
Q15056	Eukaryotic translation initiation factor 4H	3	2	6.65	3.76
Q4VXU2	Polyadenylate-binding protein 1-like	10	4	56.35	3.75
Q96A33	Coiled-coil domain-containing protein 47	2	2	11.80	3.66
P34897	Serine hydroxymethyltransferase_ mitochondrial	11	3	54.59	3.63
P06744	Glucose-6-phosphate isomerase	10	5	62.84	3.60
P35916	Vascular endothelial growth factor receptor 3	4	1	15.44	3.51
A0A087WWK8	IQ motif and SEC7 domain-containing protein 1	3	1	10.63	3.51
P07900	Heat shock protein HSP 90-alpha	47	17	347.54	3.51
P62937	Peptidyl-prolyl cis-trans isomerase A	22	12	105.92	3.50
P48449	Lanosterol synthase	8	1	45.20	3.49
P08754	Guanine nucleotide-binding protein G(k) subunit alpha	5	1	33.18	3.49
P62263	40S ribosomal protein S14	2	2	6.16	3.44
O00232	26S proteasome non-ATPase regulatory subunit 12	5	2	29.54	3.43
A0A0B4J1R6	Transketolase	10	3	63.68	3.41
Q13263	Transcription intermediary factor 1-beta	14	8	85.23	3.39
P62714	Serine/threonine-protein phosphatase 2A catalytic subunit beta isoform	4	2	25.56	3.37
Q04917	14-3-3 protein eta	8	1	76.09	3.35
Q12905	Interleukin enhancer-binding factor 2	8	4	44.31	3.34
P21912	Succinate dehydrogenase [ubiquinone] iron-sulfur subunit_ mitochondrial	12	5	59.21	3.34
P46783	40S ribosomal protein S10	2	1	14.55	3.34
P07195	L-lactate dehydrogenase B chain	13	9	74.24	3.31
B7Z9C2	cDNA FLJ58569_ highly similar to Nucleosome assembly protein 1-like 1	3	3	20.82	3.29
A0A2R8Y543	Catenin beta-1	4	1	21.70	3.28
P60174	Triosephosphate isomerase	19	11	97.12	3.26
A6NHL2	Tubulin alpha chain-like 3	3	1	30.08	3.25

Q9BUJ2	Heterogeneous nuclear ribonucleoprotein U-like protein 1	6	3	35.66	3.23
P61981	14-3-3 protein gamma	17	10	84.93	3.20
O96008	Mitochondrial import receptor subunit TOM40 homolog	4	2	27.66	3.19
O95831	Apoptosis-inducing factor 1_ mitochondrial	8	6	48.44	3.12
Q9Y536	Peptidyl-prolyl cis-trans isomerase A-like 4A	3	1	14.95	3.10
E9PLA9	Caprin-1 (Fragment)	2	2	11.22	3.10
P63104	14-3-3 protein zeta/delta	13	7	105.51	3.09
J3QS41	Probable helicase with zinc finger domain	6	1	18.61	3.08
P30086	Phosphatidylethanolamine-binding protein 1	4	3	21.02	3.02
P13637	Sodium/potassium-transporting ATPase subunit alpha-3	11	1	56.11	3.00
Q13185	Chromobox protein homolog 3	4	1	27.60	3.00
Q13885	Tubulin beta-2A chain	28	5	189.15	3.00
Q9BUP0	EF-hand domain-containing protein D1	3	2	16.60	2.98
Q15365	Poly(rC)-binding protein 1	5	1	26.47	2.97
E9PBL0	E3 ISG15--protein ligase HERC5	3	1	15.69	2.96
Q8N1F7	Nuclear pore complex protein Nup93	9	4	52.00	2.92
P22314	Ubiquitin-like modifier-activating enzyme 1	22	15	114.35	2.92
O75116	Rho-associated protein kinase 2	15	1	56.93	2.91
Q15417	Calponin-3	6	2	36.99	2.91
K7EKI0	Envoplakin	7	1	24.74	2.91
P32119	Peroxiredoxin-2	4	2	28.62	2.90
Q16658	Fascin	10	6	58.22	2.90
P07437	Tubulin beta chain	25	2	218.87	2.88
P60842	Eukaryotic initiation factor 4A-I	24	10	159.96	2.86
Q14240	Eukaryotic initiation factor 4A-II	22	5	107.25	2.86
P54652	Heat shock-related 70 kDa protein 2	19	5	110.41	2.85
P35268	60S ribosomal protein L22	3	1	16.28	2.85
A0A087WUS0	40S ribosomal protein S24	2	1	13.62	2.79
Q92598	Heat shock protein 105 kDa	8	2	42.55	2.74
P42704	Leucine-rich PPR motif-containing protein_ mitochondrial	28	11	148.59	2.72
P13639	Elongation factor 2	31	19	150.09	2.70
Q6P2Q9	Pre-mRNA-processing-splicing factor 8	8	3	36.52	2.65
P00558	Phosphoglycerate kinase 1	16	10	109.73	2.64

P00338	L-lactate dehydrogenase A chain	17	13	91.41	2.62
P68371	Tubulin beta-4B chain	32	8	201.07	2.62
P62249	40S ribosomal protein S16	7	5	32.18	2.59
P45974	Ubiquitin carboxyl-terminal hydrolase 5	7	3	32.15	2.59
P13861	cAMP-dependent protein kinase type II-alpha regulatory subunit	5	3	28.17	2.58
P30040	Endoplasmic reticulum resident protein 29	5	2	28.46	2.58
O60506	Heterogeneous nuclear ribonucleoprotein Q	20	9	134.10	2.56
P22392	Nucleoside diphosphate kinase B	9	3	51.90	2.56
Q06323	Proteasome activator complex subunit 1	9	6	54.18	2.56
P55795	Heterogeneous nuclear ribonucleoprotein H2	7	1	49.57	2.51
P55786	Puromycin-sensitive aminopeptidase	16	6	93.59	2.49
P62424	60S ribosomal protein L7a	6	4	41.53	2.49
Q86VI3	Ras GTPase-activating-like protein IQGAP3	8	1	34.67	2.48
O60716	Catenin delta-1	8	3	45.92	2.48
P63244	Receptor of activated protein C kinase 1	11	8	54.24	2.48
P62269	40S ribosomal protein S18	3	1	19.29	2.46
P23396	40S ribosomal protein S3	23	15	108.38	2.46
P05023	Sodium/potassium-transporting ATPase subunit alpha-1	14	7	78.94	2.44
P09211	Glutathione S-transferase P	10	9	92.47	2.44
P55884	Eukaryotic translation initiation factor 3 subunit B	10	5	49.98	2.42
P62753	40S ribosomal protein S6	6	4	36.89	2.39
Q99497	Protein/nucleic acid deglycase DJ-1	3	1	21.86	2.38
Q9HDC9	Adipocyte plasma membrane-associated protein	12	7	60.20	2.38
P68363	Tubulin alpha-1B chain	24	8	158.13	2.36
B4DKY1	Cysteine--tRNA ligase_ cytoplasmic	6	4	25.77	2.34
P08865	40S ribosomal protein SA	10	5	56.08	2.30
**Proteins up-regulated in MRC5 cell line**					
**Accession**	**Description**	**Peptide Count**	**Unique peptides**	**Confidence score**	**Max Fold change**
Q6P0N0	Mis18-binding protein 1	9	1	38.53	Exclusive in MRC5

P05120	Plasminogen activator inhibitor 2	5	1	29.98	Exclusive in MRC5
Q6DD88	Atlastin-3	4	2	27.77	Exclusive in MRC5
Q96SN8	CDK5 regulatory subunit-associated protein 2	5	1	22.86	Exclusive in MRC5
Q9Y678	Coatomer subunit gamma-1	5	1	21.92	Exclusive in MRC5
A0A0D9SEN1	Prolyl endopeptidase FAP	5	1	20.84	Exclusive in MRC5
H0YL12	Electron transfer flavoprotein subunit alpha_ mitochondrial (Fragment)	3	3	20.13	Exclusive in MRC5
Q99973	Telomerase protein component 1	5	1	18.10	Exclusive in MRC5
Q969X5	Endoplasmic reticulum-Golgi intermediate compartment protein 1	3	1	17.26	Exclusive in MRC5
Q16822	Phosphoenolpyruvate carboxykinase [GTP]_ mitochondrial	3	1	17.15	Exclusive in MRC5
Q6ZUX3	TOG array regulator of axonemal microtubules protein 2	3	1	17.03	Exclusive in MRC5
Q9UBR2	Cathepsin Z	3	2	16.72	Exclusive in MRC5
Q9BXU1	Serine/threonine-protein kinase 31	4	1	15.85	Exclusive in MRC5
E9PDF6	Unconventional myosin-Ib	3	1	14.99	Exclusive in MRC5
H7C1F9	Ral GTPase-activating protein subunit alpha-2 (Fragment)	4	1	13.96	Exclusive in MRC5
A0A140T9T7	Antigen peptide transporter 1	3	1	13.73	Exclusive in MRC5
A3KMH1	von Willebrand factor A domain-containing protein 8 OS=Homo sapiens OX=9606 GN=VWA8 PE=1 SV=2	4	1	13.57	Exclusive in MRC5
P53804	E3 ubiquitin-protein ligase TTC3	4	1	13.54	Exclusive in MRC5
Q9UPU5	Ubiquitin carboxyl-terminal hydrolase 24	3	1	13.30	Exclusive in MRC5
P04216	Thy-1 membrane glycoprotein	3	1	12.94	Exclusive in MRC5
Q5VZL5	Zinc finger MYM-type protein 4	3	1	12.09	Exclusive in MRC5
P22033	Methylmalonyl-CoA mutase_ mitochondrial	3	1	11.39	Exclusive in MRC5
E9PEZ1	Cullin-9	3	1	11.17	Exclusive in MRC5

A0A0C4DFN3	Monoglyceride lipase	2	1	10.40	Exclusive in MRC5
O60343	TBC1 domain family member 4	3	1	10.35	Exclusive in MRC5
Q96S97	Myeloid-associated differentiation marker	2	1	9.51	Exclusive in MRC5
P29279	Connective tissue growth factor	2	1	9.22	Exclusive in MRC5
E9PHY8	Maestro heat-like repeat-containing protein family member 1	3	1	8.97	Exclusive in MRC5
Q96AQ6	Pre-B-cell leukemia transcription factor-interacting protein 1	2	1	8.90	Exclusive in MRC5
P10253	Lysosomal alpha-glucosidase	2	1	8.65	Exclusive in MRC5
A0A0A0MT46	POU domain protein (Fragment)	2	1	7.07	Exclusive in MRC5
O60706	ATP-binding cassette sub-family C member 9	2	1	6.61	Exclusive in MRC5
Q9UHD2	Serine/threonine-protein kinase TBK1	2	1	6.54	Exclusive in MRC5
A0A0B4J2A4	3-ketoacyl-CoA thiolase_ mitochondrial	3	1	17.91	0.43
P14314	Glucosidase 2 subunit beta	12	9	62.21	0.43
Q9Y281	Cofilin-2	4	1	28.25	0.42
P36776	Lon protease homolog_ mitochondrial	13	5	62.30	0.41
Q9NYU1	UDP-glucose:glycoprotein glucosyltransferase 2	6	2	20.33	0.41
H0YDM2	Protein SOGA1 (Fragment)	2	1	7.36	0.41
O75396	Vesicle-trafficking protein SEC22b	5	2	34.44	0.41
P35913	Rod cGMP-specific 3'_5'-cyclic phosphodiesterase subunit beta	5	1	21.35	0.40
P04843	Dolichyl-diphosphooligosaccharide--protein glycosyltransferase subunit 1	14	8	88.60	0.39
Q2LD37	Transmembrane protein KIAA1109	27	2	115.64	0.39
P27708	CAD protein	14	4	75.10	0.39
P07942	Laminin subunit beta-1	11	3	62.77	0.39
H0YE29	Rho GTPase-activating protein 1 (Fragment)	3	1	14.89	0.39
O95782	AP-2 complex subunit alpha-1	11	6	70.36	0.39
O75445	Usherin	15	2	54.73	0.39

Q4KWH8	1-phosphatidylinositol 4_5-bisphosphate phosphodiesterase eta-1	19	3	67.30	0.38
P35606	Coatomer subunit beta'	15	3	75.54	0.38
Q7Z7A1	Centriolin	18	2	66.23	0.38
P07954	Fumarate hydratase_ mitochondrial	4	3	20.81	0.38
Q16643	Drebrin	3	3	21.29	0.38
P11021	Endoplasmic reticulum chaperone BiP	39	29	236.71	0.38
P42345	Serine/threonine-protein kinase mTOR	4	1	14.15	0.37
O00370	LINE-1 retrotransposable element ORF2 protein	4	2	13.17	0.37
P60709	Actin_ cytoplasmic 1	59	23	310.29	0.37
O43707	Alpha-actinin-4	45	23	312.28	0.37
P60660	Myosin light polypeptide 6	8	4	60.66	0.37
Q9ULV4	Coronin-1C	5	3	28.88	0.36
Q96K21	Abscission/NoCut checkpoint regulator	3	2	14.62	0.36
Q07864	DNA polymerase epsilon catalytic subunit A	6	1	19.99	0.36
Q5VT06	Centrosome-associated protein 350	19	5	73.38	0.36
Q8WYK1	Contactin-associated protein-like 5	8	2	29.10	0.36
Q92736	Ryanodine receptor 2	27	2	98.33	0.35
Q6KC79	Nipped-B-like protein	6	3	21.39	0.35
Q06210	Glutamine--fructose-6-phosphate aminotransferase [isomerizing] 1	6	5	32.28	0.35
P10515	Dihydrolipoyllysine-residue acetyltransferase component of pyruvate dehydrogenase complex_ mitochondrial	7	2	35.14	0.35
Q08945	FACT complex subunit SSRP1	5	2	19.99	0.35
P12814	Alpha-actinin-1	40	15	342.86	0.35
O14795	Protein unc-13 homolog B	8	1	32.27	0.35
P13674	Prolyl 4-hydroxylase subunit alpha-1	7	4	39.01	0.34
O95202	Mitochondrial proton/calcium exchanger protein	10	3	52.23	0.34
E9PEM5	Lipopolysaccharide-responsive and beige-like anchor protein	4	1	17.94	0.33
Q5VU43	Myomegalin	11	3	56.64	0.33
P06756	Integrin alpha-V	9	2	47.80	0.33
Q07954	Prolow-density lipoprotein receptor-related protein 1	13	4	60.72	0.33

P40939	Trifunctional enzyme subunit alpha_ mitochondrial	21	10	118.79	0.32
H0YLX2	DNA-binding protein RFX7	5	2	18.17	0.32
Q70UQ0	Inhibitor of nuclear factor kappa-B kinase-interacting protein	4	1	21.26	0.32
Q8IVF4	Dynein heavy chain 10_ axonemal	28	2	113.76	0.32
P04844	Dolichyl-diphosphooligosaccharide--protein glycosyltransferase subunit 2	9	5	52.62	0.32
P26641	Elongation factor 1-gamma	17	8	80.32	0.32
P68032	Actin_ alpha cardiac muscle 1	28	12	234.52	0.32
Q6S8J3	POTE ankyrin domain family member E	29	13	144.36	0.32
P23284	Peptidyl-prolyl cis-trans isomerase B	19	12	81.67	0.31
Q5T4S7	E3 ubiquitin-protein ligase UBR4	8	2	28.03	0.31
Q9NRL2	Bromodomain adjacent to zinc finger domain protein 1A	4	2	12.80	0.31
Q07065	Cytoskeleton-associated protein 4	31	26	194.90	0.31
P61158	Actin-related protein 3	6	2	36.55	0.31
Q58FF3	Putative endoplasmin-like protein	8	3	46.94	0.30
Q96QP1	Alpha-protein kinase 1	4	1	18.61	0.30
P55084	Trifunctional enzyme subunit beta_ mitochondrial	4	2	31.58	0.30
P07237	Protein disulfide-isomerase	29	26	172.99	0.29
Q8IWZ3	Ankyrin repeat and KH domain-containing protein 1	2	2	7.28	0.29
Q9H254	Spectrin beta chain_ non-erythrocytic 4	25	2	129.64	0.29
Q99536	Synaptic vesicle membrane protein VAT-1 homolog	8	6	51.74	0.29
Q08J23	tRNA (cytosine(34)-C(5))-methyltransferase	8	1	36.75	0.29
P17655	Calpain-2 catalytic subunit	5	2	34.79	0.29
Q8NHM5	Lysine-specific demethylase 2B	3	2	10.04	0.29
Q13813	Spectrin alpha chain_ non-erythrocytic 1	22	1	101.58	0.29
P27797	Calreticulin	16	9	91.14	0.28
P00505	Aspartate aminotransferase_ mitochondrial	7	3	48.92	0.28
Q9NSE4	Isoleucine--tRNA ligase_ mitochondrial	8	5	36.38	0.27
P17301	Integrin alpha-2	16	1	94.09	0.27
P61160	Actin-related protein 2	8	6	48.48	0.27

Q13724	Mannosyl-oligosaccharide glucosidase	3	1	14.65	0.27
P31040	Succinate dehydrogenase [ubiquinone] flavoprotein subunit_ mitochondrial	8	2	36.92	0.26
B9A067	MICOS complex subunit MIC60	4	2	19.96	0.26
P30048	Thioredoxin-dependent peroxide reductase_ mitochondrial	9	7	44.09	0.26
P06753	Tropomyosin alpha-3 chain	7	1	27.91	0.25
P05556	Integrin beta-1	15	13	73.91	0.24
O14578	Citron Rho-interacting kinase	19	2	81.09	0.24
O00291	Huntingtin-interacting protein 1	7	1	27.27	0.24
P09382	Galectin-1	8	6	57.86	0.23
O00203	AP-3 complex subunit beta-1	4	2	13.44	0.23
Q14247	Src substrate cortactin	7	2	39.69	0.23
P11177	Pyruvate dehydrogenase E1 component subunit beta_ mitochondrial	5	1	30.43	0.22
Q5T7B8	Kinesin-like protein KIF24	6	2	18.61	0.22
A4UGR9	Xin actin-binding repeat-containing protein 2	18	4	68.79	0.22
P07339	Cathepsin D	16	9	84.99	0.22
Q8TD26	Chromodomain-helicase-DNA-binding protein 6	9	1	30.82	0.22
O14950	Myosin regulatory light chain 12B	14	3	79.11	0.22
O60664	Perilipin-3	9	8	71.45	0.21
P09936	Ubiquitin carboxyl-terminal hydrolase isozyme L1	6	2	43.63	0.21
P15311	Ezrin	15	6	89.82	0.20
A0A087WYR3	Tumor protein D54	5	2	32.65	0.20
A0A024QZX5	Serpin B6	7	2	38.18	0.20
P35579	Myosin-9	105	65	763.97	0.20
P17813	Endoglin	3	2	15.08	0.19
Q70CQ2	Ubiquitin carboxyl-terminal hydrolase 34	7	1	27.82	0.19
Q9NZM1	Myoferlin	31	14	170.09	0.19
Q9UKA4	A-kinase anchor protein 11	3	1	7.57	0.18
P48681	Nestin	29	21	191.49	0.17
Q9Y263	Phospholipase A-2-activating protein	5	3	19.74	0.17
P31150	Rab GDP dissociation inhibitor alpha	9	2	48.79	0.17
P18031	Tyrosine-protein phosphatase non-receptor type 1	4	2	17.03	0.17
Q96HE7	ERO1-like protein alpha	4	2	20.44	0.17
O75915	PRA1 family protein 3	2	1	11.50	0.16
Q15149	Plectin	74	2	438.34	0.16

F5H365	Protein transport protein SEC23	3	1	18.43	0.16
Q2TB90	Putative hexokinase HKDC1	2	1	7.13	0.15
P02549	Spectrin alpha chain_ erythrocytic 1	21	4	66.72	0.15
A0A0G2JH68	Protein diaphanous homolog 1	2	1	7.54	0.15
Q12955	Ankyrin-3	27	6	100.27	0.15
P53634	Dipeptidyl peptidase 1	3	2	11.35	0.15
Q9NZJ4	Sacsin	30	4	124.88	0.13
Q9NQC3	Reticulon-4	9	3	59.37	0.13
Q8IWC1	MAP7 domain-containing protein 3	4	1	16.24	0.13
A0A0G2JLB3	Glucosylceramidase	3	1	19.26	0.12
P30084	Enoyl-CoA hydratase_ mitochondrial	5	3	34.43	0.12
Q7Z2Y8	Interferon-induced very large GTPase 1	17	2	63.87	0.12
O14966	Ras-related protein Rab-7L1	3	1	16.93	0.12
P00387	NADH-cytochrome b5 reductase 3	12	10	83.15	0.11
E9PRY8	Elongation factor 1-delta	5	2	37.82	0.11
P21399	Cytoplasmic aconitate hydratase	3	1	14.97	0.11
Q52LW3	Rho GTPase-activating protein 29	6	1	18.99	0.11
P16070	CD44 antigen	11	9	62.09	0.11
Q96TA1	Niban-like protein 1	7	3	43.29	0.11
P20742	Pregnancy zone protein	5	2	16.31	0.11
P08758	Annexin A5	21	17	115.48	0.11
Q8WWI1	LIM domain only protein 7	12	3	65.01	0.10
E9PCX2	Aldose reductase	3	1	19.74	0.10
Q05682	Caldesmon	11	10	67.27	0.10
H0Y9T5	m7GpppN-mRNA hydrolase (Fragment)	2	1	7.60	0.10
P26038	Moesin	23	12	175.44	0.10
Q9C0G0	Zinc finger protein 407	7	2	24.51	0.09
P27487	Dipeptidyl peptidase 4	6	3	34.26	0.09
P36957	Dihydrolipoyllysine-residue succinyltransferase component of 2-oxoglutarate dehydrogenase complex_ mitochondrial	6	2	37.49	0.09
Q96P48	Arf-GAP with Rho-GAP domain_ ANK repeat and PH domain-containing protein 1	5	1	19.02	0.09
P24844	Myosin regulatory light polypeptide 9	10	2	73.02	0.09
P17980	26S proteasome regulatory subunit 6A	4	2	19.27	0.08
C9JKI3	Caveolin (Fragment)	2	2	14.58	0.08

Q13492	Phosphatidylinositol-binding clathrin assembly protein	6	3	34.17	0.08
P61009	Signal peptidase complex subunit 3	2	1	10.96	0.08
O94925	Glutaminase kidney isoform_ mitochondrial	5	2	28.80	0.08
A0A087WV00	Diacylglycerol kinase	3	1	12.66	0.08
Q8NF91-10	Isoform 10 of Nesprin-1	5	1	23.75	0.07
P16278	Beta-galactosidase	6	4	38.74	0.07
Q8WX93	Palladin	8	3	36.60	0.06
Q96AY3	Peptidyl-prolyl cis-trans isomerase FKBP10	7	4	37.26	0.06
Q9NZN4	EH domain-containing protein 2	10	5	59.15	0.06
Q14764	Major vault protein	8	7	56.36	0.06
Q92993	Histone acetyltransferase KAT5	2	1	8.45	0.06
P08670	Vimentin	158	90	696.36	0.06
Q14164	Inhibitor of nuclear factor kappa-B kinase subunit epsilon	3	1	10.76	0.06
P49748	Very long-chain specific acyl-CoA dehydrogenase_ mitochondrial	9	7	60.87	0.05
Q9P2E9	Ribosome-binding protein 1	11	6	58.90	0.05
A0A1B0GTM3	Acid ceramidase	4	2	23.45	0.05
O43451	Maltase-glucoamylase_ intestinal	3	1	12.41	0.05
P10620	Microsomal glutathione S-transferase 1	3	2	19.85	0.05
P27105	Erythrocyte band 7 integral membrane protein	6	3	38.39	0.05
O00159	Unconventional myosin-Ic	15	8	78.78	0.05
P10619	Lysosomal protective protein	2	2	13.39	0.04
E9PP21	Cysteine and glycine-rich protein 1	3	3	19.23	0.04
F6S8M0	N-acetylglucosamine-6-sulfatase	3	3	20.12	0.04
Q562R1	Beta-actin-like protein 2	13	1	89.06	0.04
O43852	Calumenin	7	7	40.02	0.04
P11277	Spectrin beta chain_ erythrocytic	18	1	71.79	0.02
Q01995	Transgelin	25	22	154.32	0.02
Q6NZI2	Caveolae-associated protein 1	11	10	57.67	0.01
P15144	Aminopeptidase N	8	4	49.17	0.01
Q14315	Filamin-C	57	36	386.56	0.01
P43304	Glycerol-3-phosphate dehydrogenase_ mitochondrial	8	2	37.13	0.01
O95786	Probable ATP-dependent RNA helicase DDX58	2	2	6.69	0.01
O15460	Prolyl 4-hydroxylase subunit alpha-2	7	4	38.39	0.01

P17600	Synapsin-1	3	3	5.71	0.00
