# Supplementary figures and images for: Identification of Transferrin Receptor 1 (TfR1) Overexpressed in Lung Cancer Cells, and Internalization of Magnetic Au-CoFe2O4 Core-Shell Nanoparticles Functionalized with Its Ligand in a Cellular Model of Small Cell Lung Cancer (SCLC)
Source: Pharmaceutics. 2022 Aug 17;14(8):1715. doi: 10.3390/pharmaceutics14081715 (PMC9413248; doi:10.3390/pharmaceutics14081715)

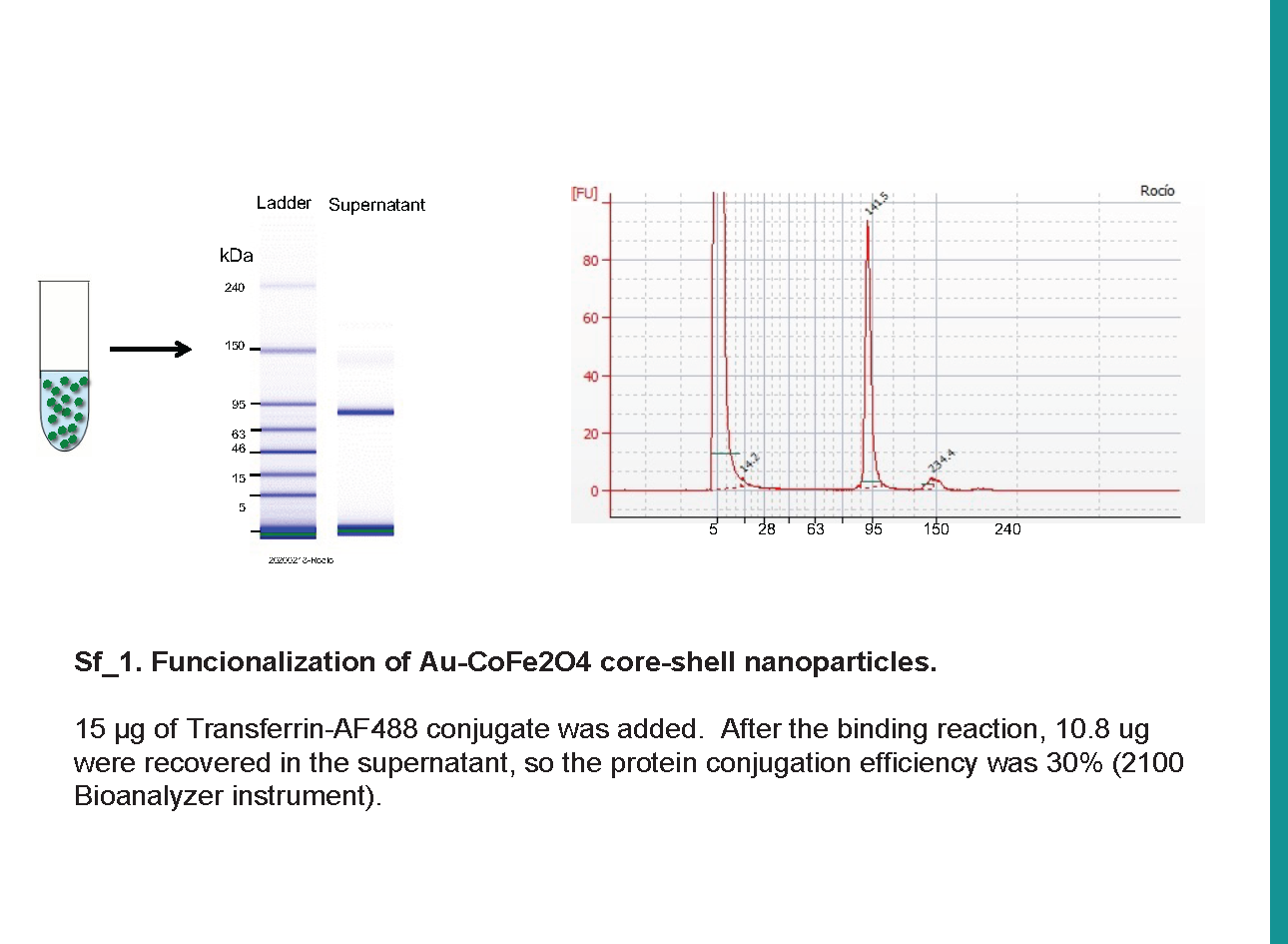

Supplement: Supplementary file 1 [file pharmaceutics-14-01715-s001.zip › SF-1 Binding eficiency TF.tiff]
